# Supplementary figures and images for: Stochastic simulations of self-organized elastogenesis in the developing lung
Source: PLoS Comput Biol. 2023 Jun 14;19(6):e1011219. doi: 10.1371/journal.pcbi.1011219 (PMC10298752; doi:10.1371/journal.pcbi.1011219)

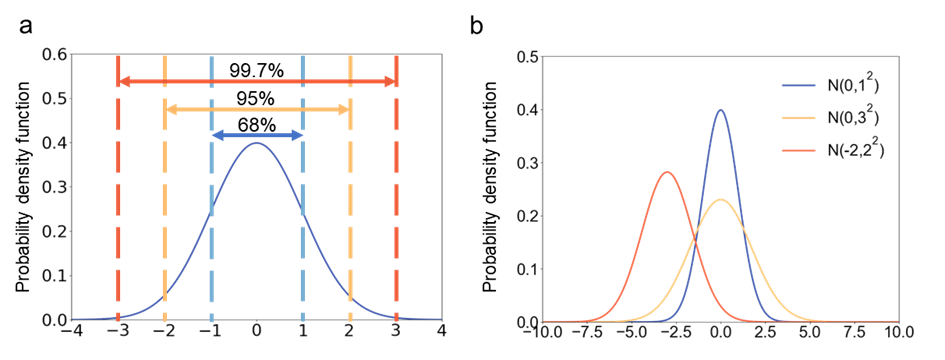

Supplement: S1 Fig — (a) The probability density function of Gaussian distribution for N(0, 12). (b) The probability density function of Gaussian distribution for N(0, 12), N(0, 32) and N(-2, 22). (TIF) [file pcbi.1011219.s001.tif]

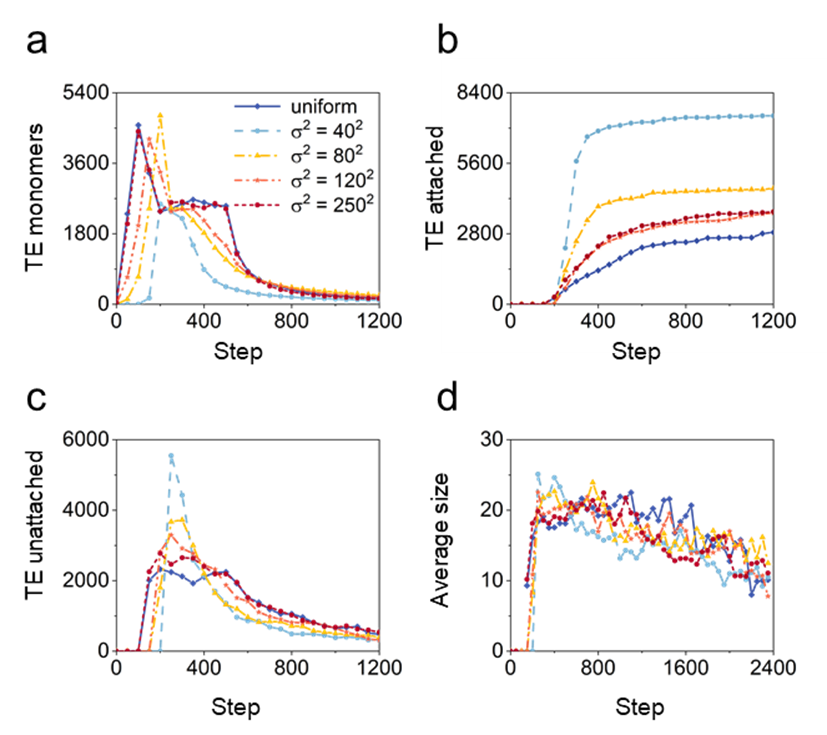

Supplement: S2 Fig — The dependence of the number of (a) TE monomers, (b) attached TE spheres, (c) unattached TE spheres, and (d) the average size of unattached TE spheres on time step with different generation time. (TIF) [file pcbi.1011219.s002.tif]

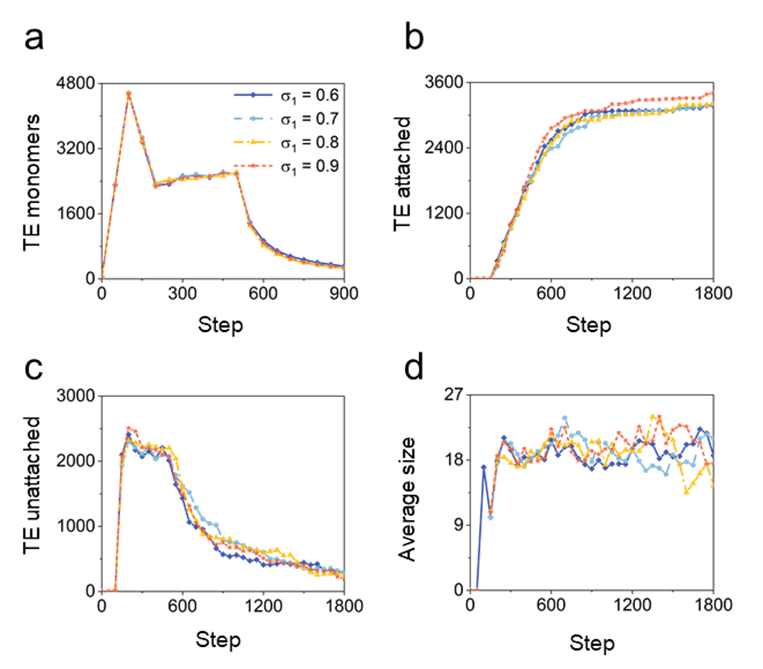

Supplement: S3 Fig — (a) total area of TE monomers; (b) total area of TEs attached to the collagen; (c) total area of TEs unattached to the collagen; (d) average area of TEs unattached to the collagen (ignore TEs smaller than 3 pixels). (TIF) [file pcbi.1011219.s003.tif]

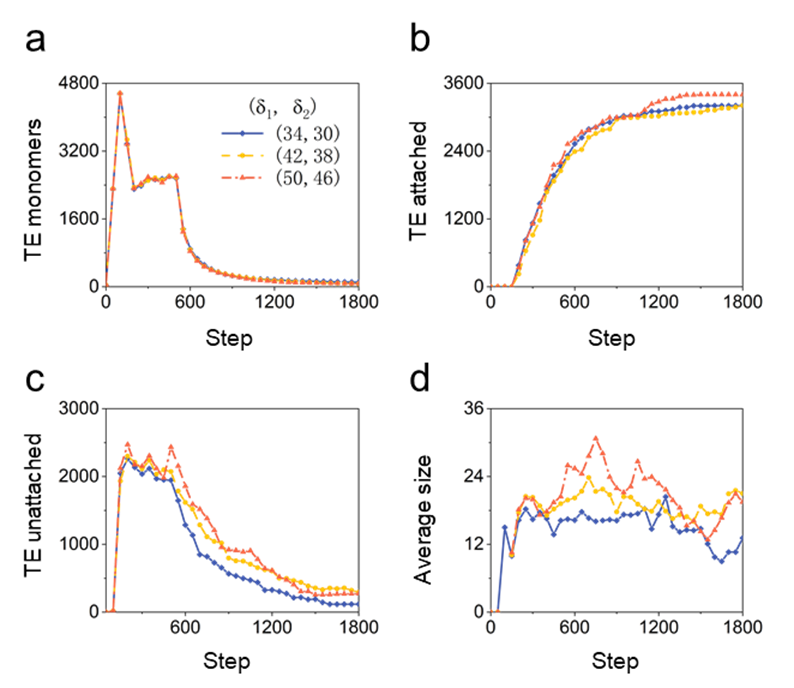

Supplement: S4 Fig — (a) total area of TE monomers; (b) total area of TEs attached to the collagen; (c) total area of TEs unattached to the collagen; (d) average area of TEs unattached to the collagen (ignore TEs smaller than 3 pixels). (TIF) [file pcbi.1011219.s004.tif]

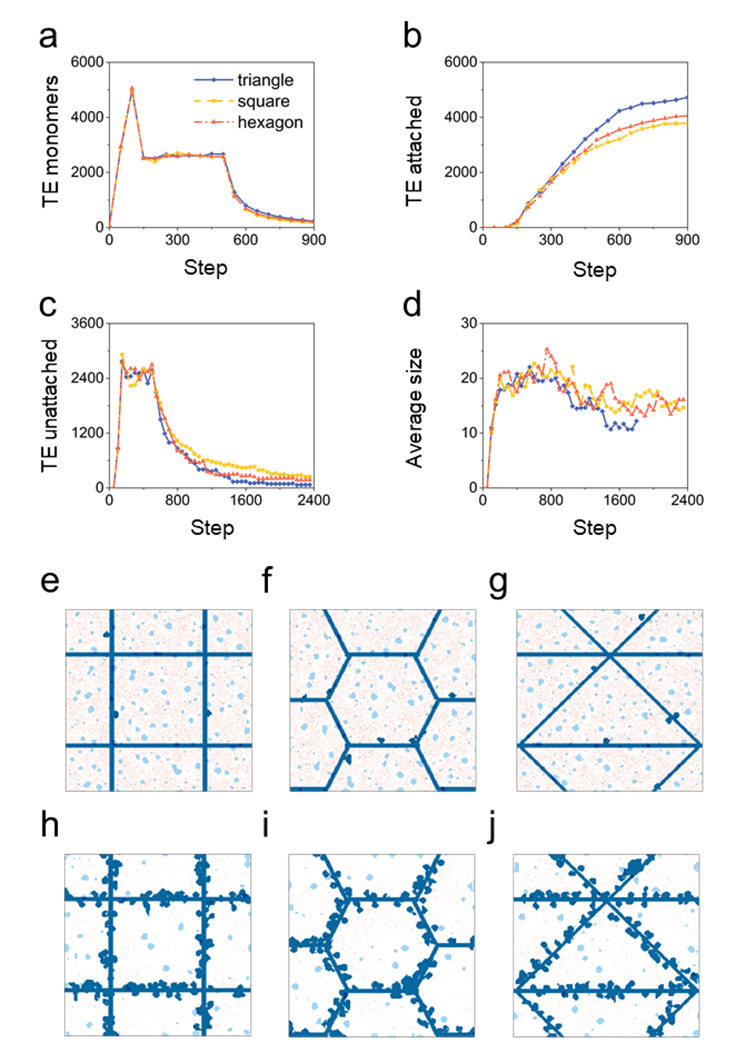

Supplement: S5 Fig — (a) TE monomers, (b) attached TE spheres, (c) unattached TE spheres, and (d) the average size of unattached TE spheres on time step with different collagen shapes. The simulation image at step (e) 250 and (h) 400 with a square collagen shape, at step (f) 250 and (i) 400 with a hexagon collagen shape and at step (g) 250 and (j) 400 with a triangle collagen shape. (TIF) [file pcbi.1011219.s005.tif]

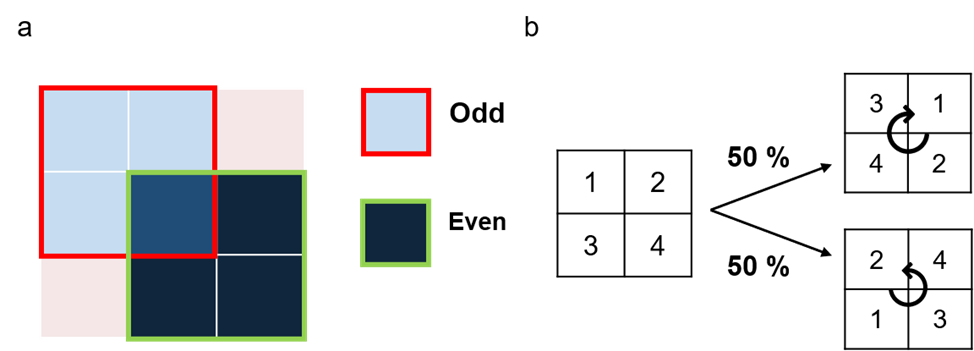

Supplement: S6 Fig — (a) The 2×2 blocks of the Margolus neighborhood; consecutive steps alternate between the even grid and the odd grid. (b) In each step, the entire block is rotated in clockwise or counterclockwise directions with equal possibilities. (TIF) [file pcbi.1011219.s006.tif]

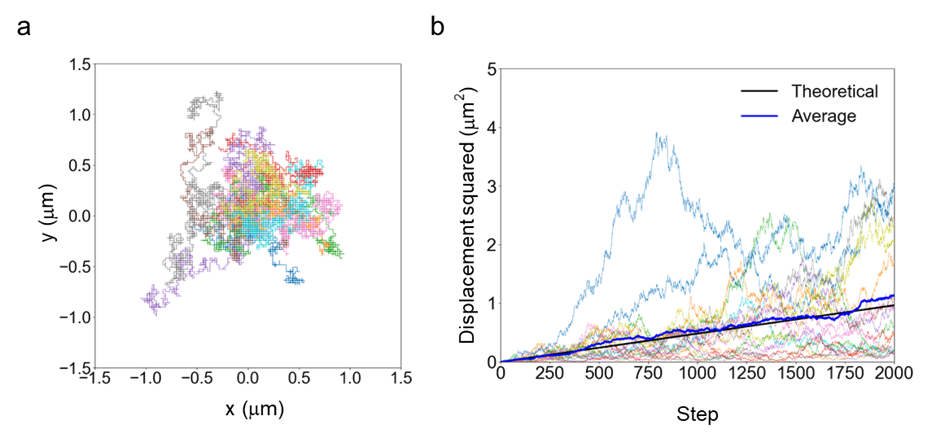

Supplement: S7 Fig — particle position trajectories. (b) The comparisons between the theoretical and average displacements squared versus time. The displacement squared of each particle is also shown. (TIF) [file pcbi.1011219.s007.tif]
