# Supplementary material for: Stochastic simulations of self-organized elastogenesis in the developing lung
Source: PLoS Comput Biol. 2023 Jun 14;19(6):e1011219. doi: 10.1371/journal.pcbi.1011219 (PMC10298752; doi:10.1371/journal.pcbi.1011219)
Supplement: Attachment — Submitted filename: PLOS Reviewer Response.docx [file pcbi.1011219.s015.docx]

Philip K Maini
Academic Editor
Jason Haugh
Section Editor
PLOS Computational Biology

RE: PCOMPBIOL-D-23-00244

Dear Editors:

Thank you for the meticulous and insightful reviews of our manuscript “Stochastic Simulations of Self-organized Elastogenesis in the Developing Lung.” We appreciate the thoughtful reviews and believe they have significantly strengthened the manuscript.

We have addressed the comments point-by-point. Please note that the substantial changes are reflected in the red font.

**Reviewer #1:**

*This work addresses the very interesting topic of how elastin cables form, placed within the context of early alveolarization in the neonatal rat lung. The authors use some innovative computational approaches to model this process from the formation of early elastin spheres into elastin cables that are required to stabilize alveoli. Overall I think its a very useful contribution.*

A: We appreciate the kind comments.

*I think it could perhaps be improved by thinking some more about what elastin is actually doing during alveologenesis. Elastin becomes organized as a set of interlinked rings that surround and stabilize the mouths of millions of alveoli.*

*When the lung is considered in 3D as opposed to 2D it is actually rather obvious that this is what is really going on as opposed to the "erection of septae" by cables lying in the "septal tips" as put forth and perpetuated by Weibel, Schittny et al. These interlocking rings also account for the elastic interdependence of the lung alveoli as well as for the failure of formation of alveoli in BPD and or in Marfan's Ehlers-Danols and other elastin matrix diseases as well as for degeneration of alveoli in emphysema. These ideas may be beyond the scope of the present paper, but I thought the authors may find them interesting to consider and perhaps incorporate into their thinking and modeling.*

A: We enthusiastically share the Reviewer’s tissue-scale insights. The key to understanding secondary septation is taking an approach that addresses 3D interdependence on a tissue scale. As suggested by the Reviewer, this is exactly where we have focused our future work.

*I have included a couple of papers FYI:*

*Conserved Mechanisms in the Formation of the Airways and Alveoli of the Lung.*

*Warburton D. Front Cell Dev Biol. 2021 Jun 15;9:662059. doi: 10.3389/fcell.2021.662059. eCollection 2021. PMID: 34211971*

*Spatial and temporal changes in extracellular elastin and laminin distribution during lung alveolar development. Luo Y, Li N, Chen H, Fernandez GE, Warburton D, Moats R, Mecham RP, Krenitsky D, Pryhuber GS, Shi W. Sci Rep. 2018 May 29;8(1):8334. doi: 10.1038/s41598-018-26673-1.*

*PMID: 29844468*

A: We appreciate the thoughtful references. We have included the references and their groundbreaking concepts in the manuscript on page 6.

**Reviewer #2**:

*This article presents a cellular automata model of the development of lung tissue. This is a geometrically simplified but illustrates that structures that may be observed in the lung could be established from cellular self-organisation, which is interesting for the general readership of this journal. However, there are some missing details in the methods making it difficult to interpret the model, and there is not a clear link regarding how this model would/could be tested against experimental data.*

*It would be useful to see a more detailed description of the line element/cable model for fibres, including reference to original studies proposing this model, or include a diagram of what is meant by a “dominant cable”. I don’t think it will be evident to all readers of this journal what is meant by a cable line element - that this is a model for the structure of the elastin fibres is not clear.*

A: We appreciate that the cable is unfamiliar with most of the readership. We have included a new Figure 1 with actual illustrations of the elastin line element.

*In the text Fig 1 is stated to show the process of structural self-organization experimentally: 1) generation of TE monomer, 2) aggregation into spheres, 3) aggregation of spheres, 4) attachment to scaffold. I cannot see this process in Fig 1. What I see in the figure is the spatial scales of importance to the process, but not the process itself. For example ,step 1 and 2 is not in the figure, the smallest scale is the spheres themselves. It is not clear which panels show step 3, I think (b/c), and then (d/e) show step 4, but this is not indicated in the caption. F. shows the model itself, but at this stage in the methods its not clear what we are seeing here, what are the lines?*

A: We have revised the new Figure 2 legend and the Discussion on page 6 accordingly.

*I think the description of the scaffold in your model is missing from the paper. You do state that your simulation is happening in a square, but I had to assume in the figures that the lines represent some sort of scaffold material. The geometry imposed on your model should be described early in the methods. Geometry is mentioned in the results but nowhere in the methods. That geometry is not clearly stated in the methods makes it difficult to then interpret the steps in the cellular automata.*

A: We regret the oversight. We have clarified the concept of the scaffold in the Introduction. We have also clarified the geometry description in the Methods on page 9.

*4.2 tropoelastin monomer: Are your constant or gaussian “generation values” distributions over time or space? How do you define the gaussian distributions (same mean as constant value? Based on some data?)?*

A: The constant or Gaussian “generation values” distributions are over time. The total generated tropoelastin monomers are the same in the simulations with Gaussian distributions and with constant generate value. We have clarified the “generation values” distributions on page 8.

*Where you discuss experimental data in the methods there should be a citation to the source of that data, are they all ref [10]?*

A: The experimental data is from [3] and [10]. We have added citations where appropriate.

*Section 4.4 “the diameters of tropoelastic spheres range from 1-6um”, section 4.6 “the size of a TE sphere is about 1 mm”. These length scales are quite different. I think this might be a typo.*

A: Corrected.

*I can see that the model is derived based on experimental data, but not so clearly the relationship back to the data on how the model could be validated or how it could guide experimental work in the future. This is hinted at in the fourth paragraph of the discussion but more explicit statements would be beneficial.*

A: We have expanded our discussion of the validation of our model in the Discussion on page 6.

*Pseudo-code is provided, I can’t see any information on, or link to, how this code was implemented, i.e. what software or tools were used*?

A: We have clarified the software and tools in the Methods on page 8. The code is written from scratch and is implemented using MATLAB (2021a).

**Reviewer #3**:

*The authors investigated the process of extracellular assembly using cellular automata (CA) simulations. The effect of the rate of the tropoelastin production on the efficiency of scaffold binding was presented. The results demonstrated that the intermediate step of tropoelastin self-aggregation into tropoelastin (TE) spheres increased the efficiency of cable formation by a fact of more than five. The rate of tropoelastin synthesis and its affinity for the protein scaffold also significantly influenced cable development, providing insight into the biophysical mechanism underlying the formation of elastin fibers in living things. The results are interesting. Following areas needs to be improved:*

*Criteria behind choosing the specific values of δ_1=42 for the division principle, and δ_2 for the crosslinking of tropoelastin and collagen scaffolds is not clear. Please elaborate.*

A: We have clarified the criteria for these values on pages 8-9.

*The selection of standard time parameters used in the simulation in Table 2 is not clear. Please discuss.*

A: The time parameters were used to reflect the biologic time course. This has been clarified on pages 9-10.

*Please provide further discussion on potential limitations or assumptions that were made in the simulations. While the results appear promising, it would be valuable to have a better understanding of the scope and limitations of the simulations to better contextualize the findings.*

A: The primary limitation was the simplification of the protein scaffold. We have clarified these points on page 6.

*Please explain the motivation behind selecting specifically three different collagen shapes, square, hexagon, and triangle. Did you change any parameters other than geometry for their simulations? Please explain how you quantified that there is no effect on the efficiency of the final scaffold by changing the geometry.*

A: The actual configuration and composition of the protein scaffold is an area of intense controversy. An objective of our work was to explore the potential impact of various geometries on elastogenesis. Interestingly, we did not find a preferential geometry to the protein scaffold.

*In Section 4.3, you mentioned two situations for the transition of tropoelastin monomers to spheres. “Both situations are based on the reasonable hypothesis that tropoelastin spheres are formed when the content of the monomers is high enough.” How high? Please be specific.*

A: The monomer critical concentration is the helical nucleation threshold; corrected in the text.

*Figure S7, sub-figures (h) (i) (j) are not detailed in the caption.*

A: We have addressed these details in Figure S7.

*Please fix the typos; e.g., “tropoleastin”*

A: Done.

We hope that these changes will be acceptable to you. Please let us know if there is anything else I can do.

Sincerely yours,

Dong Wang, PhD
